# A Repurposing Programme Evaluating Transdermal Oestradiol Patches for the Treatment of Prostate Cancer Within the PATCH and STAMPEDE Trials: Current Results and Adapting Trial Design

**DOI:** 10.1016/j.clon.2023.10.054

**Published:** 2023-11-08

**Authors:** D.C. Gilbert, M. Nankivell, H. Rush, N.W. Clarke, S. Mangar, A. Al-hasso, S. Rosen, R. Kockelbergh, S.K. Sundaram, S. Dixit, M. Laniado, N. McPhail, A. Shaheen, S. Brown, J. Gale, J. Deighan, J. Marshall, T. Duong, A. Macnair, A. Griffiths, C.L. Amos, M.R. Sydes, N.D. James, M.K.B. Parmar, R.E. Langley

**Affiliations:** * MRC Clinical Trials Unit at UCL, Institute of Clinical Trials and Methodology, London, UK; † University Hospitals Sussex NHS Foundation Trust, Royal Sussex County Hospital, Brighton, UK; ‡ The Christie and Salford Royal Hospitals, Manchester, UK; § Charing Cross Hospital, Imperial College Healthcare NHS Trust, London, UK; ǁ Beatson West of Scotland Cancer Centre, Glasgow, UK; ¶ National Heart and Lung Institute, Imperial College, London, UK; ** Department of Urology, University Hospitals of Leicester, Leicester, UK; †† Mid-Yorkshire Teaching NHS Trust, Pinderfields Hospital, Wakefield, UK; ‡‡ Scunthorpe General Hospital, Scunthorpe, UK; §§ Wexham Park Hospital, Slough, UK; ǁǁ Raigmore Hospital, Inverness, UK; ¶¶ Singleton Hospital, Swansea, UK; *** Airedale General Hospital, Keighley, UK; ††† Queen Alexandra Hospital, Portsmouth, UK; ‡‡‡ Patient Representative, MRC Clinical Trials Unit at UCL, London, UK; §§§ Guys and St Thomas’ NHS Foundation Trust, London, UK; ǁǁǁ Institute of Cancer Research, Sutton, UK

**Keywords:** Adaptive trials, androgen deprivation therapy, prostate cancer, transdermal oestrogens

## Abstract

**Aims:**

Androgen deprivation therapy (ADT), usually achieved with luteinising hormone releasing hormone analogues (LHRHa), is central to prostate cancer management. LHRHa reduce both testosterone and oestrogen and are associated with significant long-term toxicity. Previous use of oral oestrogens as ADT was curtailed because of cardiovascular toxicity. Transdermal oestrogen (tE2) patches are a potential alternative ADT, supressing testosterone without the associated oestrogen-depletion toxicities (osteoporosis, hot flushes, metabolic abnormalities) and avoiding cardiovascular toxicity, and we here describe their evaluation in men with prostate cancer.

**Materials and methods:**

The PATCH (NCT00303784) adaptive trials programme (incorporating recruitment through the STAMPEDE [NCT00268476] platform) is evaluating the safety and efficacy of tE2 patches as ADT for men with prostate cancer. An initial randomised (LHRHa versus tE2) phase II study (*n* = 251) with cardiovascular toxicity as the primary outcome measure has expanded into a phase III evaluation. Those with locally advanced (M0) or metastatic (M1) prostate cancer are eligible. To reflect changes in both management and prognosis, the PATCH programme is now evaluating these cohorts separately.

**Results:**

Recruitment is complete, with 1362 and 1128 in the M0 and M1 cohorts, respectively. Rates of androgen suppression with tE2 were equivalent to LHRHa, with improved metabolic parameters, quality of life and bone health indices (mean absolute change in lumbar spine bone mineral density of −3.0% for LHRHa and +7.9% for tE2 with an estimated difference between arms of 9.3% (95% confidence interval 5.3−13.4). Importantly, rates of cardiovascular events were not significantly different between the two arms and the time to first cardiovascular event did not differ between treatment groups (hazard ratio 1.11, 95% confidence interval 0.80−1.53; *P* = 0.54). Oncological outcomes are awaited.

**Future:**

Efficacy results for the M0 cohort (primary outcome measure metastases-free survival) are expected in the final quarter of 2023. For M1 patients (primary outcome measure – overall survival), analysis using restricted mean survival time is being explored. Allied translational work on longitudinal samples is underway.

## Introduction

Prostate cancer is the second most common cause of cancer death in men in the UK, accounting for 11700 deaths per year [1]. Although the incidence of early prostate cancer has risen dramatically through earlier detection following the introduction of prostate-specific antigen (PSA) testing, it is those cases presenting with locally advanced (M0) or metastatic disease (M1) who have the highest risk of progression and death.

Prostate cancer growth is driven by testosterone and, hence, androgen deprivation therapy (ADT) is a key component of treatment. Luteinising hormone releasing hormone analogues (LHRHa) superseded surgical castration and are the standard backbone of treatment. For M0 patients, trials such as NCIC-PR.3/MRC-PR07 [2] and EORTC-22863 [3] showed the additional benefit of radiotherapy; radiotherapy combined with 18–36 months of ADT is now a standard of care. For M1 patients, expected outcomes are not so good: data suggest a 3-year overall survival for primary presenting M1 patients of 66% [4]. In this hormone-naïve setting of *de novo* meta-static disease, the addition of upfront docetaxel (to ADT) [5,6] or one of the novel androgen receptor signalling inhibitors (ARSIs), abiraterone, apalutamide or enzalutamide, is associated with improved outcomes [7–10]. The combination of docetaxel with abiraterone improves overall survival further than docetaxel alone [11], although the use of combination ‘triplet’ therapy is not yet universally accepted.

**Fig 1:**
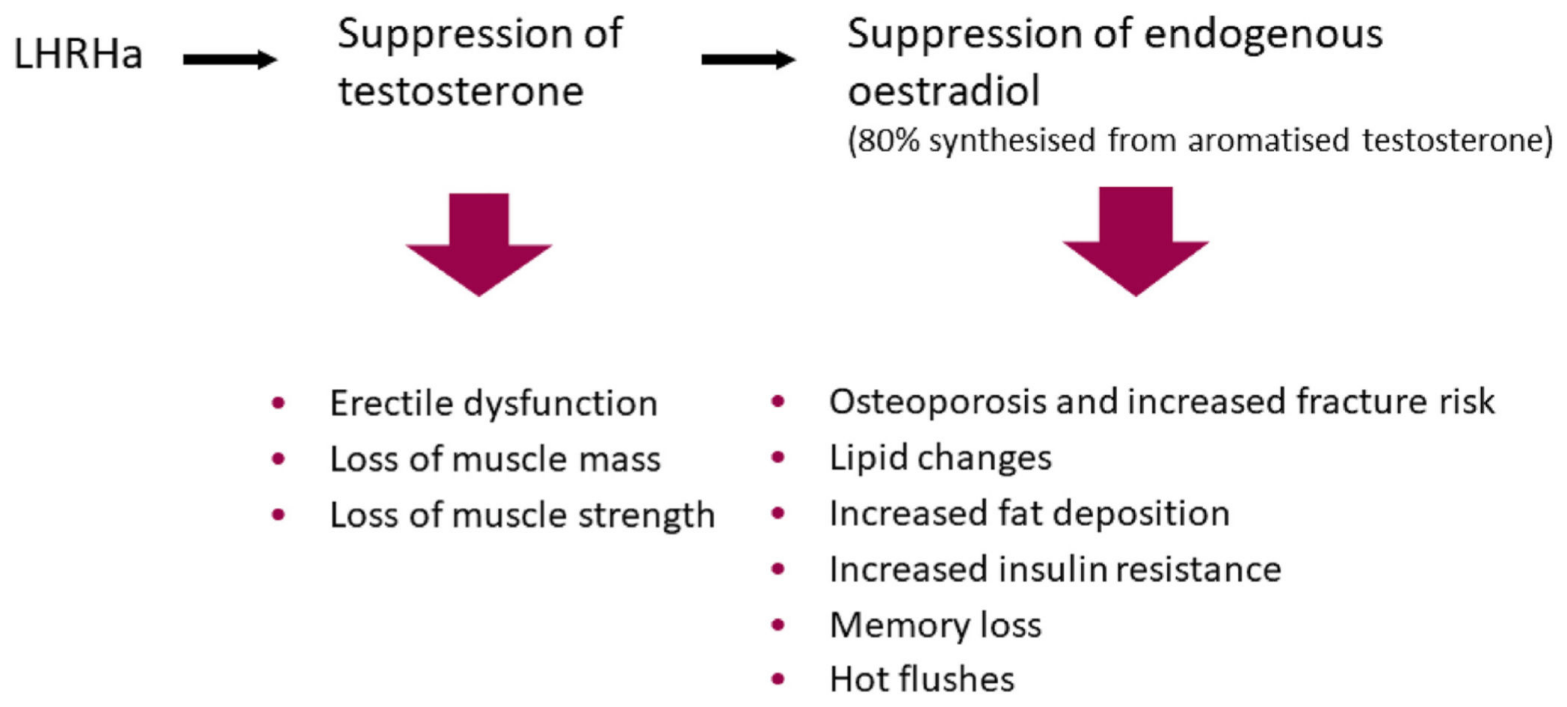
Side-effects experienced by patients undergoing androgen suppression relating to low testosterone and low oestrogenic states.

LHRHa suppress both testosterone and oestrogen (oestrogens in men are formed by the aromatisation of androgens) and this is associated with long-term deleterious effects on health, including osteoporosis, fatigue, adverse metabolic profiles and an increased risk of cardiovascular disease [12–16]. This is particularly important as over half of men diagnosed with locally advanced prostate cancer now die from other causes [17]. Oestrogens (e.g. stilboestrol) suppress testosterone production via negative feedback of LHRH production by the hypothalamus and luteinising hormone/follicular stimulating hormone by the pituitary. Oestrogens, given orally, were initially used as a method of ADT [18] but first-pass hepatic metabolism led to thrombotic complications curtailing use for this indication. Transdermal oestradiol (tE2) avoids the thrombotic toxicity and abrogates the oestrogen depletion effects of LHRHa (Figure 1), which are responsible for most of the toxicities of ADT. Therefore, tE2 offers an alternative approach for ADT with the potential long-term benefits in terms of metabolic profile, bone health and quality-of-life over LHRHa.

The evaluation of tE2 as a method of ADT in prostate cancer encompasses the PATCH (Prostate Adenocarcinoma TransCutaneous Hormones; NCT00303784) and STAMPEDE (Systemic Therapy in Advancing or Metastatic Prostate Cancer; NCT00268476) randomised controlled trials. Both were conceived to assess M0 and M1 patients as one cohort. This prospective assessment was originally a non-inferiority design on primary outcome measures of overall survival and progression-free survival (PFS). However, during the course of this work, standards of care for prostate cancer management for M0 and M1 patients have diverged, as have the associated expected prognoses. We present the rationale for and the data underpinning the evolution of the tE2 programme.

## Materials and Methods

### Trial Design and Evolution

The PATCH adaptive trials programme evaluates tE2 administered as oestradiol patches in men with M0 or M1 prostate cancer. These patches are licensed to alleviate menopausal symptoms in women. Patients eligible for the M0 cohort were those presenting with stage T3/4, N0 or NX, M0 histologically confirmed prostate adenocarcinoma with PSA ≥20 ng/ml or Gleason sum score ≥6. For the M1 cohort, men with evidence of nodal or metastatic disease or multiple sclerotic bone metastases with a PSA ≥50 ng/ml with or without histological confirmation were eligible. In addition, men previously treated with radical surgery and/or radiotherapy who were relapsing with at least one of: PSA ≥4 ng/ml and rising with doubling time less than 6 months, PSA ≥20 ng/ml or documented evidence of metastatic disease with PSA >4 ng/ml were also eligible for inclusion. Prior hormone therapy for localised disease (adjuvant or neoadjuvant) must have been completed at least 12 months previously and have been given for no longer than 12 months in duration.

Patients were randomly allocated in a 1:1 ratio to standard ADT treatment (LHRHa injections) or tE2 patches. They may have received no more than 12 weeks of an anti-androgen (typically bicalutamide) prior to randomisation to mitigate the transient increase in testosterone seen with LHRHa. Patients within STAMPEDE were eligible for the ‘tE2 comparison’ if they had received no more than a single 4-week injection of LHRHa prior to randomisation.

**Fig 2:**
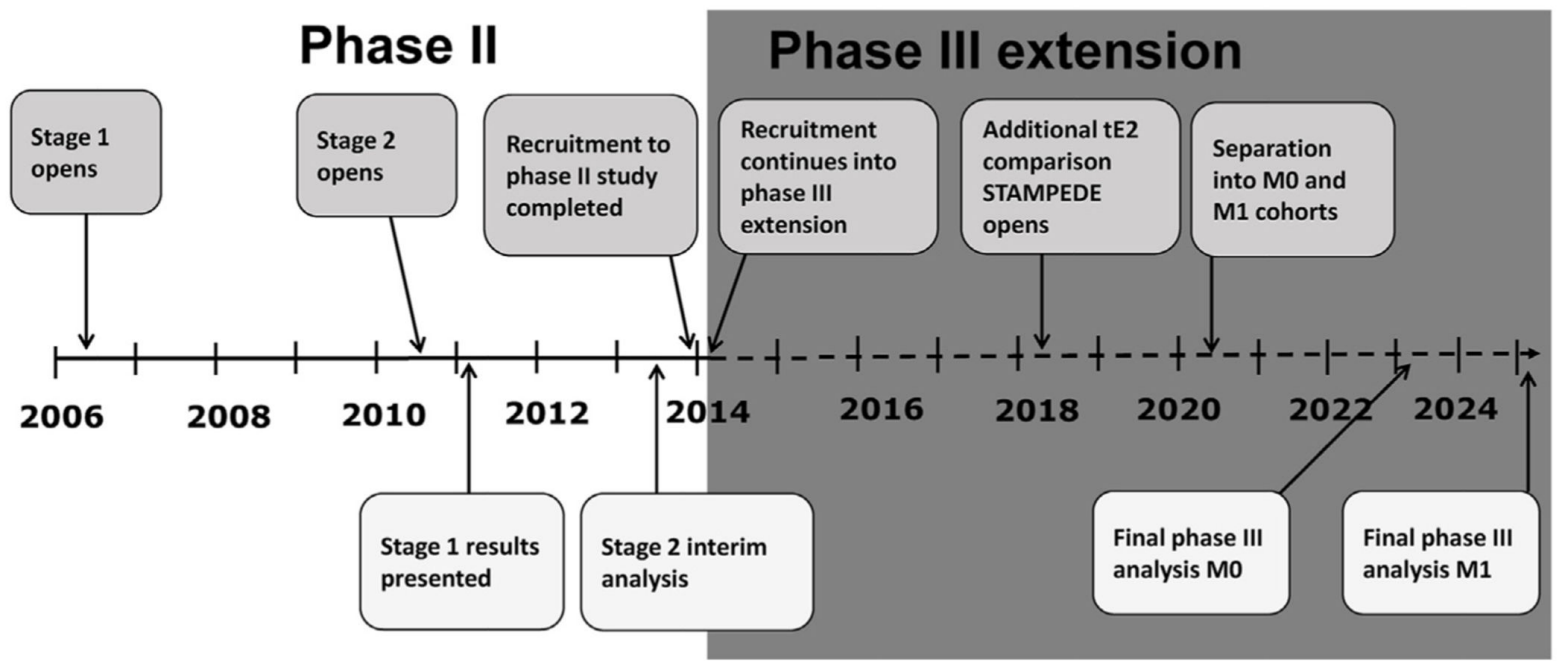
Timelines of the adaptive trials programme evaluating transdermal oestradiol (tE2) in men with prostate cancer.

The PATCH trial commenced recruitment in 2006, initially as a phase II study with cardiovascular morbidity and mortality as the primary outcome measure (Figure 2). The sample size of 251 patients was reached in March 2010. This included the first 51 patients randomised under the original patch dose regimen and established the current recommended tE2 doses [19]: induction with 4 × 100 μg Fem7 tE2 patches (Merck, KGaA, Darmstadt, Germany) until testosterone supressed and then 3 × 100 μg tE2 as maintenance. The study was then extended to recruit 680 patients (including the 200 enrolled after the dose change during the first stage) to enable a phase II evaluation of the efficacy of the patches, with a planned interim analysis performed in June 2013 based on 638 patients (stage 2). Following the stage 2 analyses, the programme was further extended in the same protocol to allow a phase III evaluation, initially planned to include a total of 2150 M0 and M1 patients.

STAMPEDE is a multi-arm, multi-stage trial, designed to simultaneously assess several treatments for men with prostate cancer [6]. STAMPEDE started recruitment in 2005 from similar populations to PATCH. As PATCH evolved into a phase III evaluation (Figure 2) it was felt that recruitment through the STAMPEDE platform was appropriate [20] and results from the two trials could be combined using a meta-analysis approach.

**Fig 3:**
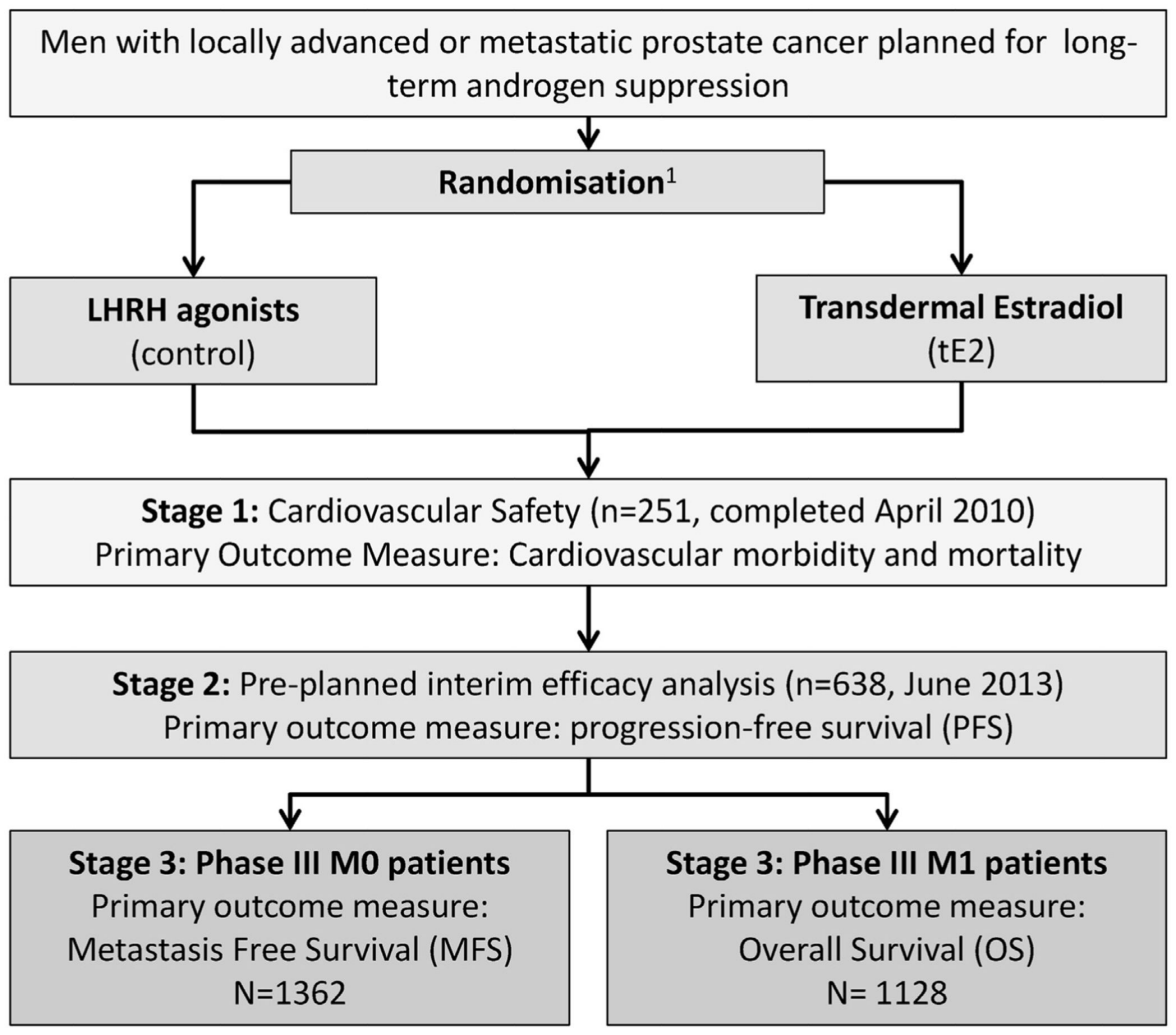
Multistage PATCH programme, with two independently powered phase III comparisons.

Reflecting changes in both management and outcomes, internationally and within the UK, driven by results from randomised controlled trials, new trials typically now evaluate patients with M0 and M1 disease separately. This has and will be followed in the STAMPEDE trial for the most recent and subsequent comparisons. To ensure that the results from the tE2 programme remain pertinent and relevant to current practice, recruitment was extended such that the M0/M1 cohorts could be individually powered at a conventional level and considered as two separate studies (Figure 3).

### Evolving Standard of Care

When PATCH was initially designed, treatment with LHRHa alone for locally advanced and metastatic patients was considered the standard of care, but recent trial results have showed increased survival through the use of additional agents alongside ADT. These have been incorporated into the tE2 programme through protocol amendments. Following the results of the NCIC-PR.3/MRC-PRO7 and SPCG-7 trials [2,3], radiotherapy was mandated for M0 patients (unless contraindicated) and as an optional treatment for M1, in later stages of the programme supported by further data from STAMPEDE [21]. Docetaxel was permitted following results from the CHAARTED and STAMPEDE trials [5,6]. Finally, with data supporting the upfront use of the ARSIs enzalutamide, abiraterone or apalutamide as alternatives to docetaxel [7–10], the protocols each permitted their use. As there was no prior experience of ARSI in combination with tE2, patients have been closely monitored to assess the safety and efficacy of this combination. Randomisation was stratified by the intention to use additional treatments to ensure they were balanced between trial arms.

### Primary Outcome Measures by Cohort

The Intermediate Clinical Endpoints in Cancer of the Prostate (ICECaP) collaboration investigated intermediate outcome measures in localised prostate cancer trials reporting that metastasis-based outcomes are usable surrogates for survival in localised prostate cancer trials where the targeted effect size is large enough [22]. For M0 patients, the primary outcome measure is now metastases-free survival (MFS), defined as the time from randomisation to confirmed metastases (excluding lymph node metastases) or death from any cause. For patients with no confirmed metastases and not known to have died, observations will be censored at the date of their most recent reported assessment. Any cases reported as a cancer-related death who have not previously reported disease progression will be reviewed by clinicians blinded to treatment allocation, with further information requested from centres, as appropriate, to ensure an earlier outcome event has not been missed. To rule out a 4% absolute detriment in 3-year MFS, from 83% in the LHRHa arm to 79% in the tE2 arm (corresponding to a hazard ratio of 1.27), with 85% power and a one-sided 5% significance level, about 510 events are required. In the original PATCH trial design, where M0 and M1 patients were to be treated as a single population, a 5% non-inferiority margin was used. Due to improvements in outcomes for M0 patients, the survival rate is now so high that it was felt patients would be less willing to accept a reduction. As such, it is felt that a smaller non-inferiority margin of 4% is more appropriate for these patients.

For M1 patients, the primary outcome measure is overall survival, defined as the time from randomisation to death from any cause. For patients not known to have died, observations will be censored at the date of their most recent reported assessment. To rule out a 5% detriment in 3-year overall survival, from 66% in the LHRH arm to 61% in the tE2 arm (corresponding to a hazard ratio of 1.19), with 80% power and a one-sided 5% significance level, about 820 events are required.

For both M0 and M1 patients, current event rates have been estimated first using the control arm patients within PATCH who did not receive radiotherapy (for M0 patients) or docetaxel (for M1 patients) and from published data for the control arm within STAMPEDE [4]. Published hazard ratios for the effect of radiotherapy and docetaxel were then applied to these estimates and combined to estimate the overall event rate for M0 and M1 patients.

### Secondary Outcome Measures

The secondary outcome measures are overall survival (for M0 patients only), PFS, prostate cancer-specific survival, cardiovascular (CVS) morbidity and mortality, CVS risk factors (including glucose and lipids), hormone levels (oestradiol, testosterone, PSA), toxicity and quality of life.

Prostate cancer-specific survival will be defined as the time from randomisation to death with the reported primary cause of death being prostate cancer. PFS will be defined as the time from randomisation to the first of three possible events: biochemical failure, clinical progression or death. For patients with no reported event at the time of analysis, observations will be censored at the date of the most recent follow-up assessment. Biochemical failure is defined according to PSA rise in respect to the PSA nadir; either a rise of 50% above the patient’s PSA nadir or a PSA rising above 4 (whichever is greater). Clinical progression is defined as any of: imaging progression, death due to prostate cancer without prior objective documentation of progression or global deterioration in health status attributable to the disease requiring a change in therapy. Progression will usually be based on PSA measurements (as above), but radiological measurements of tumour dimension/extent will take precedence over PSA response.

Quality of life is measured using patient-completed questionnaires (EORTC QLQ-C30 and PR25) at follow-up visits for 2 years post-randomisation (PATCH trial patients only).

### Statistical Analysis Plan

The final efficacy analyses will be based on data from the PATCH trial and STAMPEDE tE2 comparison, analysed separately and then combined, using a meta-analysis approach. The analysis for MFS (for M0 patients) and overall survival (for M1 patients) will take place when the required number of MFS events and deaths have been observed within the control arms across PATCH and STAMPEDE tE2. For each outcome measure, the data will be displayed using Kaplan–Meier plots and analysed with Cox regression models. If tE2 is shown to be non-inferior to LHRHa, it will then be assessed for superiority. The main analyses will be performed on an intention-to-treat basis. A secondary analysis of the primary outcome measures will focus on a per-protocol population. To take into account treatment crossover between arms, some analyses will be conducted on the subset of patients still receiving their allocated treatment at the time points of interest, for example assessments of oestradiol levels will be done among patients still receiving patches.

### Non-proportional Hazards and Restricted Mean Survival Time

Restricted mean survival time (RMST) is an alternative to using a Cox model and hazard ratios to summarise time to event outcomes. It measures average survival, effectively the area under the survival distribution, up to a prespecified time point, and is a useful alternative approach when the proportional hazards assumption does not hold [23]. In non-inferiority trials, analysis using RMST provides more power than a Cox model under nearly all circumstances, even when the proportional hazards assumption holds [24]. Recruitment to the M1 cohort, in particular, was affected significantly by the COVID-19 pandemic and subsequent challenges within the National Health Service research environment. Faced with delaying the analysis while events accrue or having to accept a reduction in power, RMST is being considered to mitigate these issues. RMST has been infrequently used as the primary analysis measure in clinical trials and we will initially assess the assumptions underlying this approach by performing a secondary analysis for the M0 cohort, evaluating MFS and overall survival using both Cox models and RMST to investigate potential gains in power and demonstrate the potential of this approach to the clinical community [24]. We will use the observed 3-year MFS rate in the control arm and the prespecified 4% absolute non-inferiority (NI) margin, and assume an exponential survival distribution, in order to translate the NI margin onto the RMST scale. In short, a change from 3-year MFS of 83%–79%, which gives the target hazard ratio of 1.265, corresponds to a change in RMST over 3 years of about 3.5 weeks. Results will inform the final analysis of the M1 cohort, with RMST potentially being used as the primary analysis method to maximise power. RMST will be assessed using a t* defined as the nearest whole year below the maximum observed follow-up time, for example if the maximum follow-up time is 10.7 years, t* will be set to 10 years.

## Results

### Recruitment to Date

Overall recruitment was paused in both PATCH and STAMPEDE during the COVID-19 pandemic that started in 2020. At this point it was decided that no further M0 patients would be recruited in either protocol, as additional patients were unlikely to contribute many events to the primary analysis. In total, 1362 M0 patients have been recruited, (which should give, in due course, 85% power to exclude a non-inferiority MFS margin of 4%) with results expected in the final quarter of 2023. For M1 patients, we retained the initial target of 5% non-inferiority margin for overall survival; recruitment continued after the early stages of the pandemic and finished in the second quarter of 2023, with 1128 patients. Efficacy analyses will occur when 822 deaths (M1 patients) have been observed, anticipated in 2024.

The multi-stage design has generated a number of stepwise results that supported the expansion of the tE2 programme to its final iteration and may provide additional important clinical considerations when the mature phase III analyses are complete.

### Castration Rates

**Fig 4:**
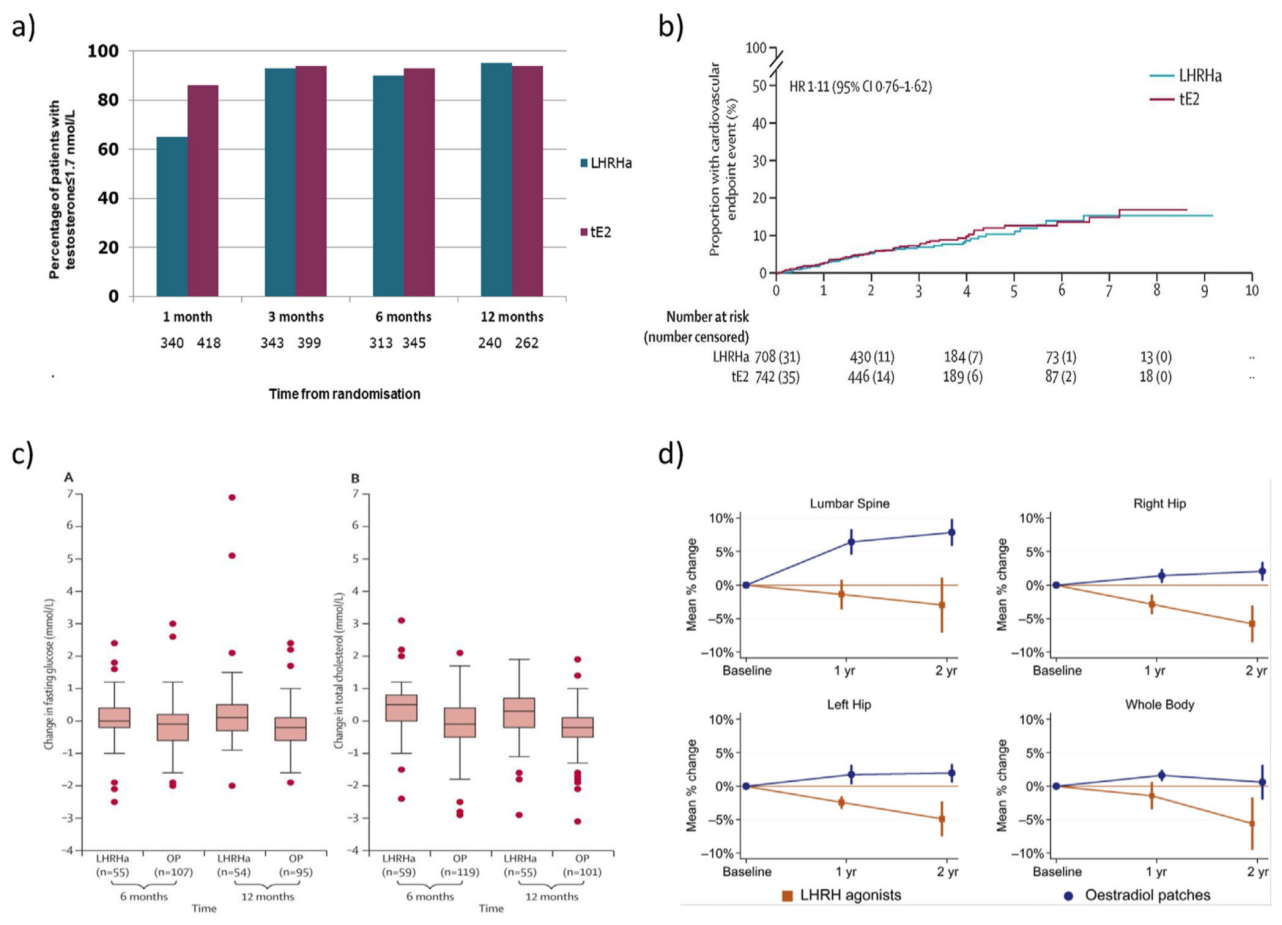
Accumulating data from the transdermal oestradiol (tE2) adaptive trials programme. (a) Rates of androgen suppression (from [25]). (b) Time to first cardiovascular end point event, intention-to-treat analysis, including patients with sudden or unexplained death and no post-mortem report events (hazard ratio 1.11, 95% confidence interval 0.80–1.53) (from [26]). (c) Changes in (A) fasting glucose and (B) total cholesterol concentrations in patients still receiving treatment at 6 and 12 months (from [25]). Boxes indicate median and interquartile range, whiskers indicate 1.5 × interquartile range; dots indicate outlying values. LHRHa, luteinising hormone releasing hormone agonists. OP, oestrogen patches. (d) Mean percentage change (95% confidence interval) in bone mineral density at 1 and 2 years from baseline by treatment arm (from [27]).

Androgen deprivation is successfully achieved to levels (of testosterone) required for the treatment of men with prostate cancer but with faster reductions with tE2 than LHRHa (Figure 4a) [25]. When rapid suppression of testosterone is required, tE2 might therefore be preferred. The high rates of rapid-onset androgen suppression support the investigation of tE2 across broader indications in men with prostate cancer with respect to shorter durations of ADT (e.g. in combination with radiotherapy in intermediate-risk prostate cancer when only 4–6 months of ADT are typically required).

### Cardiovascular Outcome Data

The initial cardiovascular outcome measures from the phase II cohort were published in 2013 [25], demonstrating equivalent numbers of cardiovascular events between tE2 and LHRHa (noting that patients with prior cardiovascular disease were excluded from the trial). Ongoing data are consistent with this finding. Most recently, following a review by the Independent Data Monitoring Committee, data from a predefined safety cohort (1694 men) over a 12-year period were published, with all predefined cardiovascular events (unstable angina/myocardial infarction, venous and arterial thromboembolic events) reviewed and verified with source data. Fifty (7%) of 708 men assigned LHRHa had a confirmed cardiovascular event as opposed to 57 (8%) of 742 of those allocated tE2. The time to first cardiovascular event did not differ between treatment groups (hazard ratio 1.11, 95% confidence interval 0.80−1.53; *P* = 0.54, Figure 4b). These definitive results confirm no excess cardiovascular toxicity from tE2 as compared with LHRHa with respect to cardiovascular events, cardiovascular deaths or time to cardiovascular event [26].

### Glucose and Lipid Profiles with tE2 versus LHRHa

An analysis of more than 800 patients recruited in PATCH showed an increase in fasting blood glucose and serum cholesterol over 12 months in patients receiving LHRHa, whereas a reduction in both levels was observed in patients treated with tE2 (12-month fasting glucose +5.9% [95% confidence interval +3.7−+8.1] in LHRHa group versus −1.1% [−2.7%, +0.6%] with tE2 [*P* < 0.0001]; 12-month fasting cholesterol +3.1% [+1.4%, +4.8%] with LHRHa versus −5.7% [−7.0%, −4.5%] with tE2 [*P* < 0.0001]; Figure 4c). Similarly, there was a statistically significant and clinically relevant difference in mean systolic and diastolic blood pressure changes at 6 months in favour of tE2 [25].

### Markers of Bone Health

Analysis of bone mineral density (BMD) measurements within PATCH confirm the hypothesis that tE2 mitigates the loss of BMD seen with LHRHa [27]. Men randomised to the tE2 arm experienced gain in BMD, with the potential to reduce long-term detrimental outcomes in terms of bone fractures with associated morbidity (Figure 4d). The mean change in lumbar spine BMD was −0.047 g/cm^3^ (mean percentage change: −3.0%) for LHRHa and +0.088 g/cm^3^ (+7.9%) for tE2 (*P* < 0.001), with an estimated difference between arms of 9.3% (95% confidence interval 5.3−13.4) (Figure 4d).

### Quality of Life

Patient-reported outcomes for 727 men enrolled in PATCH have also been published [28], demonstrating improved overall quality of life at 6 months for men on the tE2 arm. Specifically, men in the tE2 arm were less likely to experience hot flushes (8% versus 46%) and less likely to report a lack of sexual interest (59% versus 74%) and sexual activity but had higher rates of significant gynaecomastia (37% versus 5%). Potentially, the higher incidence of hot flushes among patients on LHRHa seemed to account for both the reduced global quality of life and increased fatigue as compared with tE2.

## Discussion

ADT is the cornerstone of treatment for prostate cancer, with some men remaining on ADT for many years. Optimising health and quality of life during this time has the potential for significant gains in both cancer-related and comorbid outcomes, as well as treatment compliance: hence, the programme to investigate repurposing tE2 as a method of ADT. If the results demonstrate that tE2 is not inferior to LHRHa, this will provide an alternative option for men requiring long-term androgen suppression. Potential benefits would be seen in terms of bone health (fractures are a key risk in patients treated with LHRHa [29]), metabolic profile and quality of life (particularly in a reduction in hot flushes, fatigue and sexual dysfunction). The risk of gynaecomastia is, however, increased.

As a repurposed medicine there are regulatory considerations around whether any manufacturer of tE2 patches would make an application for formal licensing for this indication [30] or whether there are alternative routes for patients to access an effective and cost-effective treatment. Patch dosing could be optimised (e.g. a formulation that would allow one patch per week at the appropriate dose for ADT). tE2 is a significantly less expensive option when compared with LHRHa; ADT represents a major component of the cost of treating men with prostate cancer [31] and prostate cancer incidence and particularly mortality continues to rise across low- and middle-income countries [32].

tE2 could also be an option for the short-term ADT used in combination with radical radiotherapy in early-stage disease. In this setting, the advantages in terms of reduced sexual dysfunction, less fatigue, etc. might be attractive for men (with the potential that the development of gynaecomastia is more limited over a shorter duration of therapy). There is scope for a clinical trial in this context, potentially with outcome measures that incorporate efficacy (adequate androgen suppression) and patient-reported factors.

The tE2 programme also represents an opportunity for ongoing translational work, aiming to understand the oncological responses to androgen suppression and, with the serial plasma and urine archive collected and stored in the early phase of the trial, studying endocrine and other metabolic changes in patients on treatment. As a large, academic trial in men with aggressive prostate cancer, these samples also provide a significant opportunity for biological insights into this disease.

## Conclusions

The tE2 programme, incorporating PATCH and STAM-PEDE, is a large phase III academic initiative investigating the repurposing of oestradiol patches as a method of androgen suppression for men with prostate cancer, with potential benefits over and above those seen with LHRHa. Recruitment is complete and a range of new data is expected in the near future, which has the potential to influence prostate cancer treatment and to help us better understand the physiological processes underpinning the biology of prostate cancer and the consequences of its treatment with ADT.

## References

[1] https://www.cancerresearchuk.org/health-professional/cancer-statistics/statistics-by-cancer-type/prostate-cancer#heading-One.

[2] Mason MD, Parulekar WR, Sydes MR, Brundage M, Kirkbride P, Gospodarowicz M (2015). Final report of the Intergroup randomized study of combined androgen-deprivation therapy plus radiotherapy versus androgen-deprivation therapy alone in locally advanced prostate cancer. J Clin Oncol.

[3] Bolla M, Van Tienhoven G, Warde P, Dubois JB, Mirimanoff RO, Storme G (2010). External irradiation with or without long-term androgen suppression for prostate cancer with high meta-static risk: 10-year results of an EORTC randomised study. Lancet Oncol.

[4] James ND, Spears MR, Clarke NW, Dearnaley DP, De Bono JS, Gale J (2015). Survival with newly diagnosed metastatic prostate cancer in the "docetaxel era": data from 917 patients in the control arm of the STAMPEDE Trial (MRC PR08, CRUK/06/019). Eur Urol.

[5] Sweeney CJ, Chen YH, Carducci M, Liu G, Jarrard DF, Eisenberger M (2015). Chemohormonal therapy in metastatic hormone-sensitive prostate cancer. N Engl J Med.

[6] James ND, Sydes MR, Clarke NW, Mason MD, Dearnaley DP, Spears MR (2016). Addition of docetaxel, zoledronic acid, or both to first-line long-term hormone therapy in prostate cancer (STAMPEDE): survival results from an adaptive, multiarm, multistage, platform randomised controlled trial. Lancet.

[7] James ND, de Bono JS, Spears MR, Clarke NW, Mason MD, Dearnaley DP (2017). Abiraterone for prostate cancer not previously treated with hormone therapy. N Engl J Med.

[8] Fizazi K, Tran N, Fein L, Matsubara N, Rodriguez-Antolin A, Alekseev BY (2019). Abiraterone acetate plus prednisone in patients with newly diagnosed high-risk metastatic castration-sensitive prostate cancer (LATITUDE): final overall survival analysis of a randomised, double-blind, phase 3 trial. Lancet Oncol.

[9] Chi KN, Agarwal N, Bjartell A, Chung BH, Pereira de Santana Gomes AJ, Given R (2019). Apalutamide for metastatic, castration-sensitive prostate cancer. N Engl J Med.

[10] Davis ID, Martin AJ, Stockler MR, Begbie S, Chi KN, Chowdhury S (2019). Enzalutamide with standard first-line therapy in metastatic prostate cancer. N Engl J Med.

[11] Fizazi K, Foulon S, Carles J, Roubaud G, McDermott R, Fléchon A (2022). Abiraterone plus prednisone added to androgen deprivation therapy and docetaxel in de novo metastatic castration-sensitive prostate cancer (PEACE-1): a multicentre, open-label, randomised, phase 3 study with a 2 × 2 factorial design. Lancet.

[12] Jespersen CG, Nørgaard M, Borre M (2014). Androgen-deprivation therapy in treatment of prostate cancer and risk of myocardial infarction and stroke: a nationwide Danish population-based cohort study. Eur Urol.

[13] Nguyen PL, Je Y, Schutz FA, Hoffman KE, Hu JC, Parekh A (2011). Association of androgen deprivation therapy with cardiovascular death in patients with prostate cancer: a meta-analysis of randomized trials. JAMA.

[14] D’Amico AV, Denham JW, Crook J, Chen MH, Goldhaber SZ, Lamb DS (2007). Influence of androgen suppression therapy for prostate cancer on the frequency and timing of fatal myocardial infarctions. J Clin Oncol.

[15] Carneiro A, Sasse AD, Wagner AA, Peixoto G, Kataguiri A, Neto AS (2015). Cardiovascular events associated with androgen deprivation therapy in patients with prostate cancer: a systematic review and meta-analysis. World J Urol.

[16] Bosco C, Bosnyak Z, Malmberg A, Adolfsson J, Keating NL, Van Hemelrijck M (2015). Quantifying observational evidence for risk of fatal and nonfatal cardiovascular disease following androgen deprivation therapy for prostate cancer: a meta-analysis. Eur Urol.

[17] Chowdhury S, Robinson D, Cahill D, Rodriguez-Vida A, Holmberg L, Møller H (2013). Causes of death in men with prostate cancer: an analysis of 50,000 men from the Thames Cancer Registry. BJU Int.

[18] Turo R, Tan K, Thygesen H, Sundaram SK, Chahal R, Prescott S (2015). Diethylstilboestrol (1 mg) in the management of castration-resistant prostate cancer. Urol Int.

[19] Langley RE, Godsland IF, Kynaston H, Clarke NW, Rosen SD, Morgan RC (2008). Early hormonal data from a multicentre phase II trial using transdermal oestrogen patches as first-line hormonal therapy in patients with locally advanced or metastatic prostate cancer. BJU Int.

[20] Gilbert DC, Duong T, Sydes M, Bara A, Clarke N, Abel P (2018). Transdermal oestradiol as a method of androgen suppression for prostate cancer within the STAMPEDE trial platform. BJU Int.

[21] Parker CC, James ND, Brawley CD, Clarke NW, Ali A, Amos CL (2022). Radiotherapy to the prostate for men with metastatic prostate cancer in the UK and Switzerland: long-term results from the STAMPEDE randomised controlled trial. PLoS Med.

[22] Xie W, Regan MM, Buyse M, Halabi S, Kantoff PW, Sartor O, ICECaP Working Group (2017). Metastasis-free survival is a strong surrogate of overall survival in localized prostate cancer. J Clin Oncol.

[23] Royston P, Parmar MK (2013). Restricted mean survival time: an alternative to the hazard ratio for the design and analysis of randomized trials with a time-to-event outcome. BMC Med Res Methodol.

[24] Quartagno M, Morris TP, Gilbert DC, Langley RE, Nankivell MG, Parmar MK (2023). A comparison of different population-level summary measures for randomised trials with time-to-event outcomes, with a focus on non-inferiority trials. Clin Trial.

[25] Langley RE, Cafferty FH, Alhasso AA, Rosen SD, Sundaram SK, Freeman SC (2013). Cardiovascular outcomes in patients with locally advanced and metastatic prostate cancer treated with luteinising-hormone-releasing-hormone agonists or transdermal oestrogen: the randomised, phase 2 MRC PATCH trial (PR09). Lancet Oncol.

[26] Langley RE, Gilbert DC, Duong T, Clarke NW, Nankivell M, Rosen SD (2021). Transdermal oestradiol for androgen suppression in prostate cancer: long-term cardiovascular outcomes from the randomised Prostate Adenocarcinoma Transcutaneous Hormone (PATCH) trial programme. Lancet.

[27] Langley RE, Kynaston HG, Alhasso AA, Duong T, Paez EM, Jovic G (2016). A randomised comparison evaluating changes in bone mineral density in advanced prostate cancer: luteinising hormone-releasing hormone agonists versus transdermal oestradiol. Eur Urol.

[28] Gilbert DC, Duong T, Kynaston HG, Alhasso AA, Cafferty FH, Rosen SD (2017). Quality-of-life outcomes from the Prostate Adenocarcinoma: TransCutaneous Hormones (PATCH) trial evaluating luteinising hormone-releasing hormone agonists versus transdermal oestradiol for androgen suppression in advanced prostate cancer. BJU Int.

[29] Parry MG, Cowling TE, Sujenthiran A, Nossiter J, Berry B, Cathcart P (2019). Identifying skeletal-related events for prostate cancer patients in routinely collected hospital data. Cancer Epidemiol.

[30] https://www.england.nhs.uk/wp-content/uploads/2021/03/B0342-oportunities-to-repurpose-medicines-in-the-nhs-in-england.pdf.

[31] Sangar VK, Ragavan N, Matanhelia SS, Watson MW, Blades RA (2005). The economic consequences of prostate and bladder cancer in the UK. BJU Int.

[32] Wang L, Lu B, He M, Wang Y, Wang Z, Du L (2022). Prostate cancer incidence and mortality: global status and temporal trends in 89 countries from 2000 to 2019. Front Public Health.

